# A Double-Activity (Green Algae Toxicity and Bacterial Genotoxicity) 3D-QSAR Model Based on the Comprehensive Index Method and Its Application in Fluoroquinolones’ Modification

**DOI:** 10.3390/ijerph17030942

**Published:** 2020-02-03

**Authors:** Lu-ze Yang, Miao Liu

**Affiliations:** Department of Chemistry, College of New Energy and Environment, Jilin University, Changchun 130012, China; yanglz19@mails.jlu.edu.cn

**Keywords:** fluoroquinolones, comprehensive index method, 3D-QSAR, green algae toxicity, bacterial genotoxicity

## Abstract

The comparative molecular similarity index analysis (CoMSIA) model of double-activity quinolones targeting green algae toxicity and bacterial genotoxicity (8:2) was constructed in this paper on the basis of the comprehensive index method. The contour maps of the model were analyzed for molecular modifications with high toxicities. In the CoMSIA model, the optimum number of components *n* was 7, the cross-validated *q*^2^ value was 0.58 (>0.5), the standard deviation standard error of estimate (SEE) was 0.02 (<0.95), *F* was 1265.33, and the non-cross-validated *R*^2^ value was 1 (>0.9), indicating that the model had a good fit and predicting ability. The scrambling stability test parameters *Q*^2^, cross-validated standard error of prediction (cSDEP), and d*q*^2^/d*r*^2^yy were 0.54, 0.25, and 0.8 (<1.2), respectively, indicating that the model had good stability. The external verification coefficient *r*^2^_pred_ was 0.73 (>0.6), and standard error of prediction (SEP) was 0.17, indicating that the model had a good external prediction ability. The contribution rates of the steric fields, electrostatic fields, hydrophobic fields, hydrogen bond donor, and acceptor fields were 10.9%, 19.8%, 32.7%, 13.8%, and 22.8%, respectively. Large volume groups were selected for modification of ciprofloxacin (CIP), and the derivatives with increased double-activity characterization values were screened; the increase ratio ranged from 12.31–19.09%. The frequency of derivatives were positive and total energy, bioaccumulation, and environmental persistence was reduced, indicating that the CIP derivatives had good environmental stability and friendliness. Predicted values and CoMSIA model constructed of single activities showed that the CoMSIA model of double activities had accuracy and reliability. In addition, the total scores of the derivatives docking with the D1 protein, ferredoxin-NADP (H) reductases (FNRs), and DNA gyrase increased, indicating that derivatives can be toxic to green algae by affecting the photosynthesis of green algae. The mechanism behind the bactericidal effect was also explained from a molecular perspective.

## 1. Introduction

Quinolones are a class of synthetic antibiotics [1] that have been divided into four generations according to the order of invention and antibacterial effect. Fluoroquinolones (FQs) encompass the third generation (including norfloxacin, ofloxacin, ciprofloxacin, etc.) and the fourth generation (including levofloxacin, gatifloxacin, moxifloxacin, etc.) quinolones that are used widely because of their good pharmacokinetic properties and therapeutic effects in the treatment of bacterial infections in humans and animals [2]. FQs have become one of the most widely used antibiotics in the world [3]. The metabolization and absorption of FQs in both human and animal bodies are not strong, and thus about 30–70% of the FQs ingested through oral administration or injection are expelled into the natural environment [4,5], and a large number of unused expired FQs (including FQs in medical waste water) [6] have been discharged into urban sewage. Antibiotics may contaminate surface water, drinking water, and groundwater sources. Although most urban sewage goes through sewage treatment systems, the removal efficiency of antibiotics in typical sewage treatment plants is only at about 60–90% [7]. High concentrations of FQs have been detected in rivers found at the center of China’s Guangdong Province [8], Beijing’s Wenyu River [9], and Taihu Lake [10], and the concentration ranged from 80.9–3148 ng/L.

In recent years, a large amount of nitrogen and phosphorus nutrients generated by intense human activities have entered urban lakes as a result of the rapid development of urbanization, thereby accelerating the process of eutrophication in water bodies and causing water blooms [11]. Over the past four decades, green macroalgae blooms large enough to result in green tides have been occurring with greater frequency in coastal areas worldwide. These blooms negatively affect the ecology and economy of coastal environments [12]. These water blooms cause lake water bodies to deteriorate significantly in a short time, causing the water to become black and smelly and thus leading to the death a large number of fishes. Quinolones selectively inhibit DNA gyrase and topoisomerase IV, which play important roles in DNA synthesis, thus interfering with the replication, transcription, and repair of bacterial DNA and inhibiting bacteria from passage, which are collectively defined as genotoxicity [13]. The results of the present study suggest that even low ciprofloxacin (CIP) concentrations can affect the growth of aquatic photoautotrophs [14]. It is important to study the role of residual quinolones in water, especially in the control of water blooms.

This paper attempted to build a comparative molecular similarity index analysis (CoMSIA) model of double-activity quinolones for both green algae toxicity and bacterial genotoxicity on the basis of the comprehensive index method. FQ derivatives with increased double toxic activities were designed on the basis of the contour maps of the CoMSIA model, and the environmental friendliness and stability of the derivatives were evaluated. Finally, the derivatives with increased green algae toxicity, enhanced bactericidal ability, environmental friendliness, and environmental stability were screened out to generate some ideas for the treatment of algae blooms.

## 2. Materials and Methods

### 2.1. Data Sources of Quinolones’ Toxicity

Green algae toxicity and bacterial genotoxicity data of 29 quinolones and 21 FQ derivatives with low biological enrichment [15], high photodegradation [16], and low ADRs (adverse drug reactions) [17] were obtained to construct the CoMSIA model. Green algae toxicity data were retrieved from the EPIWEB 4.1 database (https://www.epa.gov/, EPA, Washington, D.C., USA), represented by EC_50_, and bacterial genotoxicity data of quinolones and FQ derivatives were derived from the literature [18] and predicted by hologram quantitative structure-activity relationship (HQSAR) model established by Zhao et al. [13], respectively, as represented by the lowest observed effective concentration (LOEC).

### 2.2. Calculation of Double-Activity Characterization Values of Green Algae Toxicity and Bacterial Genotoxicity on the Basis of the Comprehensive Index Method

The comprehensive index method converts multiple index values of the evaluation object into a comprehensive index through the corresponding statistical processes to achieve an overall evaluation [19]. The double-activity characterization values of green algae toxicity and bacterial genotoxicity of quinolones and FQ derivatives were calculated using the comprehensive index method, and the formula is as follows:

(1) $$Y=\frac{X}{M}$$

Z = 0.8Y_1_ + 0.2Y_2_ (2)

where Y is the indexed indicator of green algae toxicity (Y_1_) and bacterial genotoxicity (Y_2_) of quinolones and FQ derivatives, X is the value of green algae toxicity/bacterial genotoxicity, and M is the standard value of green algae toxicity/bacterial genotoxicity, which is expressed as mean value of each single toxicity. After indexing the two toxicity indices, they were integrated according to a ratio of 8:2; Z is the double-activity characterization value of green algae toxicity and bacterial genotoxicity of quinolones and FQ derivatives.

### 2.3. Construction Method of the CoMSIA Model of the Double-Activity Quinolones’ Green Algae Toxicity and Bacterial Genotoxicity

The CoMSIA model was constructed using the double-activity characterization value of green algae toxicity and bacterial genotoxicity of quinolones and FQ derivatives as the dependent variable; the independent variable was the molecular structure parameters. Using SYBYL-X 2.0 software (Tripos Inc., Saint Louis, MO, USA) to draw molecular structures, the Minimize module was applied. The electrical charges contained in the molecules were Gasterger–Hückle charges. The Tripos molecular force field was used, and the energy convergence standard was 0.005 kcal/mol and iteration was 10,000, thereby allowing the molecules to reach the most stable conformation. The optimized molecular common skeleton was superimposed, and the molecule with the highest double-activity characterization value was selected as the template molecule.

Fourty molecules were randomly selected as the training set and the remainder acted as the test set to establish the CoMSIA model for the double activities of green algae toxicity and bacterial genotoxicity using SYBYL-X 2.0 software [20]. The partial least squares method was applied, and the leave-one-out method was used for cross-validation of the training set’s compounds to obtain the cross-validation coefficient *q*^2^ and the optimal principal component number *n*. Regression analysis was carried out through No validation, and *R*^2^, standard deviation standard error of estimate (SEE), test value *F*, and the contribution rates of force fields (steric, electrostatic, hydrophobic, hydrogen bond receptor, and donor) were calculated. The scrambling stability test was constructed to determine the robustness of the model. The evaluation parameters were *Q*^2^, cross-validated standard error of prediction (cSDEP) and d*q*^2^/d*r*^2^yy. Cross-validation was used to test the external prediction ability of the model, and the evaluation parameters standard error of prediction (SEP) and *r*^2^_pred_ were calculated. Finally, the contour maps of the constructed CoMSIA model were analyzed to determine the positions and properties of the substituted groups.

### 2.4. Environmental Stability Evaluation Method of FQ Derivatives on the Basis of Gaussian Calculations

The frequencies and total energies were calculated using Gaussian 09 software (Gaussian Inc., Wallingford, CT, USA) [21] on the basis of the density functional theory at the level of B3PW91/6-31G* to evaluate the environmental stability of the derivatives [22]. A positive frequency indicates that the derivatives can stably exist in the environment [23], and a lower energy indicates a better stability [24].

### 2.5. Environmentally Friendly Evaluation Method for FQ Derivatives on the Basis of EPI

The predicted values of bioaccumulation and environmental persistence were obtained from the EPIWEB 4.1 database using the simplified molecular input line entry system (SMILES) number of the FOs derivatives as expressed by log*K_ow_* and t_1/2_(river), respectively, to evaluate the environmental friendliness of the derivatives.

### 2.6. Mechanism Analysis of Green Algae Toxicity and Bacterial Genotoxicity Based on Molecular Docking

The LibDock module in the Discovery Studio 4.0 software (BIOVIA Inc., Shenzhen, China) was applied to dock the target molecule and its derivatives with proteins that represent green algae toxicity and bacterial genotoxicity, respectively, to evaluate the mechanisms for the increase of two toxicities after molecular modification. The binding energies of the derivatives docking with the proteins were calculated to verify the accuracy of the molecular docking.

## 3. Results and Discussion

### 3.1. Construction and Evaluation of the CoMSIA Model of Double-Activity Quinolones’ Green Algae Toxicity and Bacterial Genotoxicity

#### 3.1.1. Calculation of Double-Activity Characterization Values of Quinolones’ Green Algae Toxicity and Bacterial Genotoxicity

**Figure 1:**
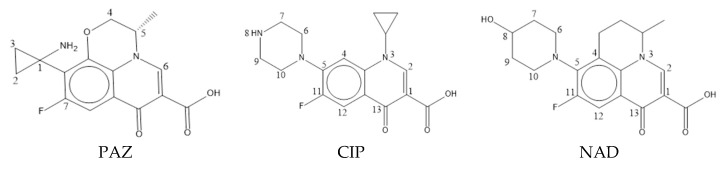
Molecular structure of pazufloxacin (PAZ), ciprofloxacin (CIP), and nadifloxacin (NAD).

Table 1 lists the quinolones and FQ derivatives’ green algae toxicity (*pEC*_50_), bacterial genotoxicity (*pLOEC*), and the double-activity characterization values calculated using the comprehensive index method at a ratio of 8:2. The higher the characterization value, the higher the double activities of both green algae toxicity and bacterial genotoxicity. As shown in Table 1, moxifloxacin (MOX) had the highest double-activity characterization value; therefore, MOX was selected as the template molecule to construct the CoMSIA model. The molecular structure of pazufloxacin (PAZ), ciprofloxacin (CIP), and nadifloxacin (NAD) are shown in Figure 1.

#### 3.1.2. Construction of the CoMSIA Model of the Double-Activity Quinolones’ Green Algae Toxicity and Bacterial Genotoxicity

The evaluation parameters of the constructed CoMSIA model of quinolones’ green algae toxicity and bacterial genotoxicity (8:2) are shown in Table 2. In the CoMSIA model, the optimum number of components *n* was 7, and the cross-validated *q*^2^ value was 0.58 (>0.5), indicating that the model had a good predicting ability. The standard deviation SEE was 0.02 (<0.95), *F* was 1265.33, and the non-cross-validated *R*^2^ value was 1 (>0.9), indicating that the model had a good fitting ability. The external verification coefficient *r*^2^_pred_ was 0.73 (>0.6), and SEP was 0.17, indicating that the model had a good external prediction ability. The scrambling stability test parameters *Q*^2^, cSDEP, and d*q*^2^/d*r*^2^yy were 0.54, 0.25, and 0.8 (<1.2), respectively, indicating that the model had good stability [25]. The force fields that influenced the model were the electrostatic fields (E, 19.8%), steric fields (S, 10.9%), hydrophobic fields (H, 32.7%), hydrogen bond donor fields (D, 13.8%), and hydrogen bond acceptor fields (A, 22.8%) (Table 3).

#### 3.1.3. Contour Map Analysis of the CoMSIA Model for Double-Activity Quinolones’ Green Algae Toxicity and Bacterial Genotoxicity

**Figure 2:**
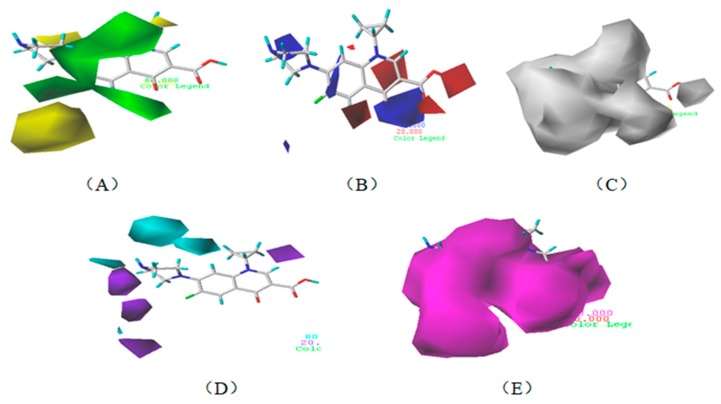
Contour maps of the CoMSIA model’s steric fields (**A**), electrostatic fields (**B**), hydrophobic fields (**C**), hydrogen bond donor fields (**D**), and hydrogen bond acceptor fields (**E**).

Figure 2 shows the contour maps of the constructed CoMSIA model. The CIP molecule was taken as an example. The hydrophobic and hydrogen bond acceptor fields had a higher contribution rate; the white (Figure 2C) and purple area (Figure 2E) covered almost the entire molecule, indicating that they had effects on each site and were not representative. As the contour maps of the electrostatic fields (Figure 2B) show, the blue area was distributed at the C=O position of the carbonyl group and at the N atom position of piperazine ring. The red area was mainly distributed at site 13 and the common skeleton of quinolones. Regarding the contour map of the hydrogen bond donor fields, the colors were both mainly distributed around the piperazine ring, which had no significant effects. The steric fields are shown in Figure 2A; the green area distributed at site 3 and around the benzene ring, and the yellow area was mainly around the piperazine ring. The introduction of the large volume group in the green area or small volume group in the yellow area would enhance the double-activity characterization value of green algae toxicity and bacterial genotoxicity of the CIP molecule [26]. In summary, the substitution of large volume substituent groups at site 3 would improve both the green algae toxicity and bacterial genotoxicity of the CIP molecule.

### 3.2. Molecular Design of CIP Derivatives with Enhanced Double-Activity FQs’ Green Algae Toxicity and Bacterial Genotoxicity

According to the contour map information taken from the constructed CoMSIA model, three substituents with a larger volume than -C_3_H_5_ were selected for substitution at site 3 of the CIP molecule, and three derivatives with enhanced double-activity characterization values of green algae toxicity and bacterial genotoxicity were obtained (Table 4). The increased rates of the double-activity characterization values of the derivatives ranged from 12.31–19.09%, among which the highest increase was the derivate substituted by -CH_2_-C_3_H_5_ at site 3 of the CIP molecule. The volume of the -CH_2_-C_3_H_4_Cl and -CH_2_-C_3_H_5_ group was larger than that of the -CH_2_CH_2_CH_3_ group, and the characterization value of the CIP derivative substituted by the former was stronger than that of the latter, indicating that the results were consistent with the contour maps’ information on the CoMSIA model. Previously, Aruoja et al. [27], Chen et al. [28], and Huang et al. [29] established QSAR models of green algae toxicity of anilines, phenols, and nitriles, respectively. In addition, Miguel et al. [30] and Ebert et al. [14] examined the green algae toxicity of five and two FQs, respectively. No systematic studies on the quinolone green algae toxicity have been found. The QSAR model that was established in this paper combined the comprehensive index method, and, for the first time, can analyze the green algae toxicity of quinolones, and the model also contained the genotoxicity information simultaneously, which was innovative.

### 3.3. Environmental Stability of the CIP Derivatives on the Basis of Gaussian Calculations

The calculation results for frequency and total energy of the CIP derivatives are shown in Table 5. The frequencies of the derivatives were all greater than 0, indicating that the derivatives could exist stably in the environment. Compared to the target molecule, the total energy of four designed derivatives decreased at a range of 0.11–43.45%, indicating that the derivatives were more stable, among which the no. 2 (3-CH_2_-C_3_H_4_Cl-CIP) derivative had higher stability.

### 3.4. Environmentally Friendly Evaluation of CIP Derivatives on the Basis of EPI

The bioaccumulation and environmental persistence of the designed CIP derivatives were evaluated using EPIWEB 4.1 (Table 6). The change rates of the bioaccumulation of derivatives were in the range of −32.14% to 139.29%, whereas the change rates were reduced in the no. 3 (3-CH_2_CH_2_CH_3_-CIP) derivatives. The change rates in the environmental persistence of derivatives were −55.98% to 129.02% compared to the CIP molecule, whereas the change rates were reduced in the no. 1 (3-CH_2_-C_3_H_5_-CIP) and no. 3 (3-CH_2_CH_2_CH_3_-CIP) derivatives. In summary, the no. 3 (3-CH_2_CH_2_CH_3_-CIP) derivative was recognized as the best derivative in terms of environmental friendliness (bioaccumulation, environmental persistence).

### 3.5. Validation of Double-Activity Qunolones’ Toxicities in the CoMSIA Model

#### 3.5.1. Validation of the CoMSIA Model on the Basis of the Single Activities of Green Algae Toxicity and Bacterial Genotoxicity

The EPIWEB 4.1 database and the HQSAR model established by Zhao et al. were used to predict the green algae toxicity and bacterial genotoxicity of the derivatives, and the change rates of these two toxic activities were calculated (Table 7). The green algae toxicity and bacterial genotoxicity of derivatives increased at a range of 25.195–60.70% and 23.26–31.77%, respectively. For the same derivative, the increased rate of green algae toxicity was significantly higher than that of bacterial genotoxicity. The CoMSIA model was established using logarithmic level data; thus, the ratio of two logarithmic level toxicity changes were calculated. The ratio of increased rates between green algae toxicity and bacterial genotoxicity of the 3-CH2-C3H5-CIP derivative was 4.55, which was essentially consistent with the ratio of the two toxic activities (8:2) that was set to construct the CoMSIA model. This indicated that the model had some accuracy and could be effectively used in the FQ molecular selective modification of green algae toxicity and bacterial genotoxicity.

#### 3.5.2. Reliability Analysis of the CoMSIA Model on the Basis of the Contour Maps of a Single Activity of Green Algae Toxicity and Bacterial Genotoxicity

To verify whether the constructed CoMSIA model of the double activities encompassed the single activity characteristics of green algae toxicity and bacterial genotoxicity, the CoMSIA models for these two activities were built. Table 8 lists the CIP molecule contour maps of the molecular force fields of both double and single activity CoMSIA models. The contribution rates of the relevant force fields are marked under each contour map. The orders of the contribution rates of the molecular force fields in the three models were basically the same; they were expressed as hydrogen bond acceptor fields and hydrophobic fields had higher contribution rates. By comparing the contour maps of the steric fields of the three constructed models, the situation at site 3 and piperazine ring was similar. The electrostatic fields and hydrogen bond donor fields of the double-activity model were partially congruent with the two single activity models. In the hydrophobic fields of the double-activity and green algae toxicity models, the grey area basically covered the entire molecule, whereas in the bacterial genotoxicity model, it distributed at site 4 and 8. There were only purple areas in the three contour maps of the hydrogen bond acceptor fields, and the double-activity model contained the information of the single-activity models. In summary, the double-activity model was similar to the partial force fields of the single activity models, indicating that the established double-activity model has reliability.

### 3.6. Mechanism Analysis of Green Algae Toxicity and Bacterial Genotoxicity on the Basis of Molecular Docking

#### 3.6.1. Molecular Docking of the CIP Derivatives with the D1 Protein and DNA Gyrase

The D1 protein is an important constituent protein of photosystem II (PSII) in chloroplasts [31] and is the most sensitive protein in response to various stress conditions among the four hydrophobic transmembrane proteins that make up PS II [32,33,34]. The main targets for the antibacterial action of FQs are DNA gyrase (Gram-negative bacteria) and topoisomerase IV (Gram-positive bacteria) [35]. The bacterial genotoxicity data used herein was derived from Gram-negative bacteria. The structure of D1 protein and DNA gyrase were downloaded from the Protein Data Bank (PDB) protein database (http://www.rcsb.org/); the PDB IDs were 1FC6 and 5Z9P, respectively. The total scores of the CIP molecule and its derivatives docking with these two proteins are shown in Table 9.

The total scores of the CIP derivatives docking with the D1 protein increased by 8.1–14.83% compared to the CIP molecule, indicating that the derivatives were more likely to bind to the D1 protein to affect photosynthesis, as photosynthesis is an important process for plants to generate energy. The no. 2 (3-CH_2_-C_3_H_4_Cl-CIP) molecule had the highest total score (Table 9) and green algae toxicity (Table 7), indicating that derivatives can theoretically bind to the D1 protein, thereby causing a toxic effect on green algae.

The total scores of the CIP derivatives docking with DNA gyrase increased by 13.44–19.72%, and the trend was similar to the prediction results of bacterial genotoxicity, as the no. 3 (3-CH_2_CH_2_CH_3_-CIP) derivative had a relatively small bacterial genotoxicity and total score. The results show that the derivatives were more easily combined with DNA gyrase than the CIP molecule, thereby exerting the bactericidal effect of FQs.

**Figure 3:**
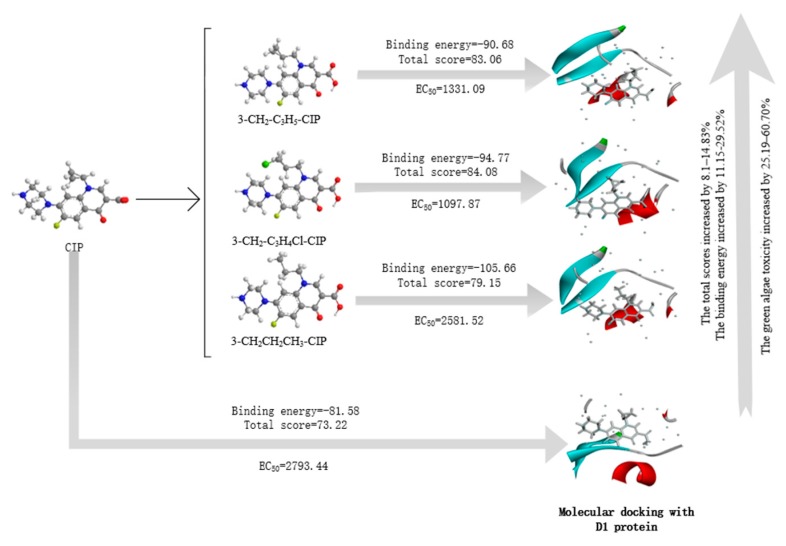
The mechanism of CIP derivatives’ green algae toxicity.

Taking green algae toxicity as an example, the binding energy of the derivatives to the protein was calculated as shown in Table 9. The binding energy of all the derivatives was increased, indicating that they were easier to bind to the protein. The binding energy change rates of the no. 1 (3-CH_2_-C_3_H_5_-CIP) and no. 2 (3-CH_2_-C_3_H_4_Cl-CIP) molecules were basically consistent with their change in total scores. The accuracy of the molecular docking scores was verified. The mechanism of CIP derivatives’ green algae toxicity is shown in Figure 3.

#### 3.6.2. Molecular Docking of the CIP Derivatives with Ferredoxin-NADP (H) Reductases (FNRs)

The ferredoxin-NADP (H) reductases (FNRs) [36] that also affect photosynthesis were chosen to dock with the derivatives in order to further study the mechanism of the derivatives’ green algae toxicity. The structure of FNRs was downloaded from the PDB protein database and the PDB ID was 1FC6. The total scores of CIP derivatives docking with FNRs (Table 9) increased by 8.58–16.15%, being consistent with the results of the derivatives docking with the D1 protein, indicating that the CIP derivatives can be toxic to green algae by adjusting photosynthesis.

## 4. Conclusions

A CoMSIA model for double-activity quinolones’ green algae toxicity and bacterial genotoxicity was constructed, and model verification allowed it to be successfully applied to the CIP modification. Three CIP molecule derivatives with increased green algae toxicity and bacterial genotoxicity were designed and screened. The derivatives were stable and environmentally friendly, thereby providing new ideas for multi-activity modification of pollutants to control water blooms.

## Figures and Tables

**Table 1:** Green algae toxicity, bacterial genotoxicity, and double-activity characterization values of quinolones.

No.	Name	*pEC*_50_	*pLOEC*	Composite Value
1	Pipemidic acid (PIP)	−4.59	4.80	−0.94
2 *	Cinoxacin (CIN)	−2.24	6.52	−0.17
3	Norfloxacin (NOR)	−3.64	7.67	−0.48
4 *	Balofloxacin (BAL)	−2.83	7.31	−0.28
5	Ciprofloxacin (CIP)	−3.45	7.85	−0.41
6	Danofloxacin (DAN)	−3.17	7.66	−0.35
7 *	Difloxacin (DIF)	−2.64	8.06	−0.17
8	Enrofloxacin (ENR)	−3.00	8.75	−0.23
9	Fleroxacin (FLE)	−3.46	6.77	−0.49
10	Gatifloxacin (GAT)	−3.16	8.28	−0.30
11	Lomefloxacin (LOM)	−3.26	7.54	−0.38
12	Moxifloxacin (MOX)	−0.32	8.89	0.53
13	Nadifloxacin (NAD)	−1.62	8.08	0.11
14	Ofloxacin (OFL)	−3.62	7.96	−0.45
15 *	Pazufloxacin (PAZ)	−3.52	8.54	−0.38
16	Rufloxacin (RUF)	−3.55	6.89	−0.51
17	Sarafloxacin (SAR)	−2.77	7.92	−0.22
18 *	Sparfloxacin (SPA)	−3.44	7.37	−0.44
19	Levofloxacin (LEV)	−3.62	7.75	−0.47
20 *	Enoxacin (ENO)	−3.58	7.08	−0.50
21	Pefloxacin (PEF)	−3.51	7.96	−0.42
22 *	Amifloxacin (AMI)	−4.39	8.00	−0.66
23 *	Besifloxacin (BES)	−2.43	7.50	−0.15
24	Clinafloxacin (CLI)	−3.08	7.19	−0.36
25	Grepafloxacin (GRE)	−2.81	7.47	−0.26
26	Marbofloxacin (MAR)	−4.26	8.46	−0.60
27 *	Orbifloxacin (ORB)	−2.67	7.51	−0.22
28 *	Sitafloxacin (SIT)	−2.69	7.28	−0.24
29	Temafloxacin (TEM)	−2.38	8.47	−0.07
30	1-CH_3_-PAZ	−0.98	8.05	0.29
31	1-H-PAZ	−1.27	7.67	0.18
32	5-OH-PAZ	−4.87	8.38	−0.77
33	5-F-PAZ	−3.90	7.57	−0.56
34	1-C_2_H_3_-5-F-PAZ	−1.14	7.13	0.18
35	1-CO-5-OH-PAZ	−3.39	7.72	−0.41
36	1-CO-5-F-PAZ	−2.42	7.14	−0.18
37	7-OH-CIP	−4.53	8.25	−0.69
38	7-C_2_H_5_-CIP	−2.85	7.98	−0.24
39	7-CN-CIP	−3.86	8.07	−0.51
40	7-NO-CIP	−3.66	8.14	−0.45
41	7-OCH_3_-CIP	−3.68	8.38	−0.44
42	7-C_2_H_3_-CIP	−2.94	8.34	−0.24
43	7-COOH-CIP	−4.70	8.31	−0.73
44	9-F-NAD	−1.73	8.33	0.10
45	9-Cl-NAD	−1.53	8.32	0.15
46	9-Br-NAD	−1.52	8.28	0.15
47	2-C_2_H_3_-NAD	−1.02	8.32	0.29
48	2-C_2_H_3_-9-F-NAD	−1.13	8.18	0.25
49	2-C_2_H-9-F-NAD	−1.57	8.29	0.14
50	2-C_2_H_3_-9-Cl-NAD	−1.37	8.29	−0.17

* represents the test set of the CoMSIA model, the remainder are the training set. *pEC*_50_—green algae toxicity; *pLOEC*—bacterial genotoxicity.

**Table 2:** Evaluation parameters of the CoMSIA model based on the characterization values of double-activity quinolones’ green algae and bacterial genotoxicity.

Model	*q* ^2^	*n*	SEE	*R* ^2^	*F*	*r* ^2^_pred_	SEP	*Q* ^2^	cSDEP	d*q*^2^/d*r*^2^yy
CoMSIA	0.58	7	0.02	1	1265.33	0.73	0.17	0.54	0.25	0.8

*q*^2^—cross-validated value; *n*—the optimum number of components; SEE—standard error of estimate; *R*^2^—non-cross-validated value; *r*^2^_pred_—external verification coefficient; SEP—standard error of prediction; cSDEP—cross-validated standard error of prediction; d*q*^2^/d*r*^2^yy—the slope of Q^2^ with respect to the correlation of the original dependent variables against the perturbed dependent variables.

**Table 3:** Molecular fields’ contribution to the characterization values of double-activity quinolones’ green algae and bacterial genotoxicity estimated by the CoMSIA model.

Model	S	E	H	D	A
CoMSIA	10.9%	19.8%	32.7%	13.8%	22.8%

S—steric fields; E—electrostatic fields; H—hydrophobic fields; D—hydrogen bond donor fields; A—hydrogen bond acceptor fields.

**Table 4:** Prediction of double-activity characterization values of the CIP derivatives’ green algae and bacterial genotoxicity.

No.	Molecule	Comprehensive Value	Change Rate of Comprehensive Value (%)
0	CIP	−0.604	-
1	3-CH_2_-C_3_H_5_-CIP	−0.489	19.09
2	3-CH_2_-C_3_H_4_Cl-CIP	−0.529	12.47
3	3-CH_2_CH_2_CH_3_-CIP	−0.530	12.31

**Table 5:** Calculation values of frequency and total energy of the CIP derivatives.

No.	Molecule	Frequency	Total Energy	Change Rate of Total Energy
0	CIP	26.78	−1147.95	-
1	3-CH_2_-C_3_H_5_-CIP	25.79	−1187.27	3.43
2	3-CH_2_-C_3_H_4_Cl-CIP	18.25	−1646.78	43.45
3	3-CH_2_CH_2_CH_3_-CIP	20.12	−1149.18	0.11

**Table 6:** Bioaccumulation and environmental persistence of the CIP derivatives.

No.	Molecule	log*K_ow_*	Change Rate of log*K_ow_*	t_1/2_ (River)	Change Rate of t_1/2_ (River)
0	CIP	0.28	-	8.72 × 10^13^	-
1	3-CH_2_-C_3_H_5_-CIP	0.49	75	6.71 × 10^13^	−23.12
2	3-CH_2_-C_3_H_4_Cl-CIP	0.67	139.29	2.00 × 10^14^	129.02
3	3-CH_2_CH_2_CH_3_-CIP	0.19	−32.14	3.84 × 10^13^	−55.98

**Table 7:** Green algae toxicity, bacterial genotoxicity, and their enhancement ratio for CIP derivatives.

No.	Molecule	EC_50_	LOEC	Change Rate of EC_50_ (%)	Change Rate of LOEC (%)	The Ratio of Two Toxicity Changes (Logarithmic Level)
0	CIP	2793.44	1.40 × 10^−8^	-	-	-
1	3-CH_2_-C_3_H_5_-CIP	1331.09	9.66 × 10^−9^	52.35	30.98	4.55
2	3-CH_2_-C_3_H_4_Cl-CIP	1097.87	9.55 × 10^−9^	60.70	31.77	5.56
3	3-CH_2_CH_2_CH_3_-CIP	2581.52	1.07 × 10^−8^	25.19	23.26	0.67

**Table 8:** The contour maps of the CoMSIA models for both the single activity and double activities of green algae toxicity and bacterial genotoxicity.

Force Field	CoMSIA Model of Double-Activity	CoMSIA Model of Green Algae Toxicity	CoMSIA Model of Bacterial Genotoxicity
Steric	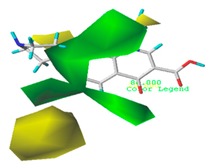 10.8%	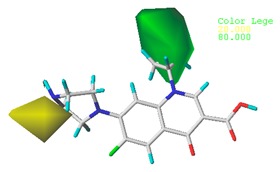 4.1%	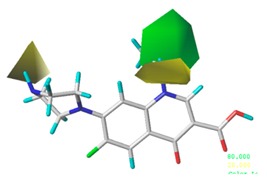 7.8%
Electrostatic	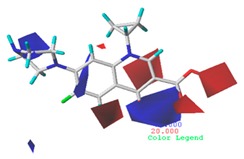 19.8%	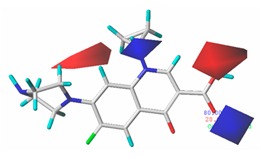 13.7%	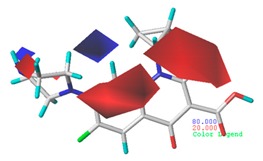 17.7%
Hydrophobic	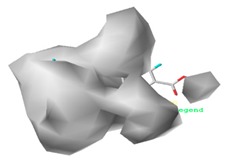 32.7%	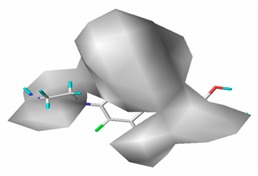 26.1%	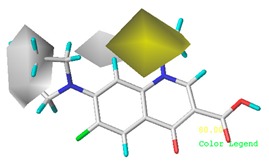 21.3%
Donor	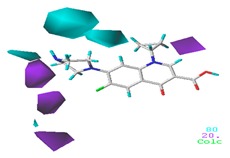 13.8%	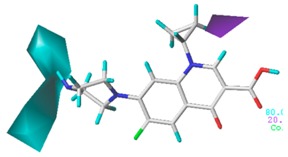 5.4%	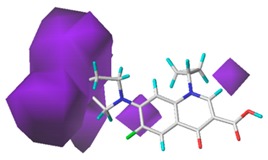 4.4%
Acceptor	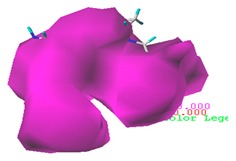 22.8%	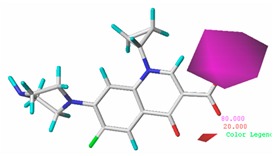 50.7%	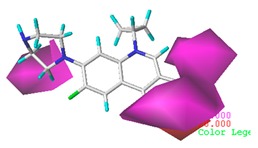 48.7%

**Table 9:** The molecular docking score of the CIP derivatives.

No.	Molecule	Total Score (1FC6)	Change Rate	Binding Energy	Change Rate	Total Score (5Z9P)	Change Rate	Total Score (2XNC)	Change Rate
0	CIP	73.22	-	−81.58	-	59.22	-	71.08	-
1	3-CH_2_-C_3_H_5_-CIP	83.06	13.44	−90.68	11.15	70.89	19.72	77.82	9.48
2	3-CH_2_-C_3_H_4_Cl-CIP	84.08	14.83	−94.77	16.17	69.01	16.54	82.56	16.15
3	3-CH_2_CH_2_CH_3_-CIP	79.15	8.1	−105.66	29.52	67.18	13.44	77.17	8.58
